# Mathematical Modelling of p53 Signalling during DNA Damage Response: A Survey

**DOI:** 10.3390/ijms221910590

**Published:** 2021-09-30

**Authors:** Ján Eliaš, Cicely K. Macnamara

**Affiliations:** 1 Institute of Mathematics and Scientific Computing, University of Graz, Heinrichstrasse 36, 8010 Graz, Austria; 2 School of Mathematics and Statistics, Mathematical Institute, University of St Andrews, St Andrews KY16 9SS, UK

**Keywords:** p53 models, oscillatory dynamics, negative feedback, time delays, spatial models, positive feedback

## Abstract

No gene has garnered more interest than p53 since its discovery over 40 years ago. In the last two decades, thanks to seminal work from Uri Alon and Ghalit Lahav, p53 has defined a truly synergistic topic in the field of mathematical biology, with a rich body of research connecting mathematic endeavour with experimental design and data. In this review we survey and distill the extensive literature of mathematical models of p53. Specifically, we focus on models which seek to reproduce the oscillatory dynamics of p53 in response to DNA damage. We review the standard modelling approaches used in the field categorising them into three types: time delay models, spatial models and coupled negative-positive feedback models, providing sample model equations and simulation results which show clear oscillatory dynamics. We discuss the interplay between mathematics and biology and show how one informs the other; the deep connections between the two disciplines has helped to develop our understanding of this complex gene and paint a picture of its dynamical response. Although yet more is to be elucidated, we offer the current state-of-the-art understanding of p53 response to DNA damage.

## 1. Introduction

The term gene regulatory network (GRN) is used mathematically to describe, by way of a graph/network, any collection of genetic molecules (nodes) which mutually interact (edges). Interactions typically affect changes to the production of the molecules through promotion and inhibition mechanisms [1]. GRNs are found ubiquitously across all cellular mechanisms and processes and are, as such, of great importance and interest to those seeking to unravel cell behaviour. Perhaps more importantly, GRNs are a vital tool in understanding what happens, on a genetic level, when cells deviate from their typical behaviour, during the onset of cancer, for example. A key player in molecular oncology is p53 [2,3,4,5,6], known simply as the tumour suppressor protein it is frequently mutated in cancer [7,8]. The protein(s) p53, derived from the *TP53* gene in humans provide many regulatory functions. Specifically, its role in healthy cells is to suppress changes to a cancerous phenotype by gatekeeping key cellular processes such as apoptosis and cell cycle arrest [9,10,11]. It is for this reason that p53 has also gained the title of *guardian of the genome* [12]. Mathematicians have been interested in modelling the p53 genetic pathway for over 20 years. In this review we will direct our attention to the efforts of the mathematical biology community in modelling GRNs and specifically those involving p53 in order to provide an up to the moment discussion of our understanding of the p53 genetic pathway.

Extra- and inter-cellular stresses (for example, heat, DNA damage and hypoxia) activate p53 [11,13]; its subsequent behaviour and downstream affects depend on the nature of the stress [14,15,16]. Most experiments have focussed on activation of p53 by irradiation, which causes DNA double stranded breaks. Double stranded breaks lead to phosphorylation of p53 which affects its binding to its main antagonist Mdm2 leading to an increase in p53 [14]. The subsequent dynamical behaviour of p53 has been the focus of most studies both from an experimental and mathematical point of view. In this review we will focus primarily on the response of p53 to DNA damage.

**Figure 1:**
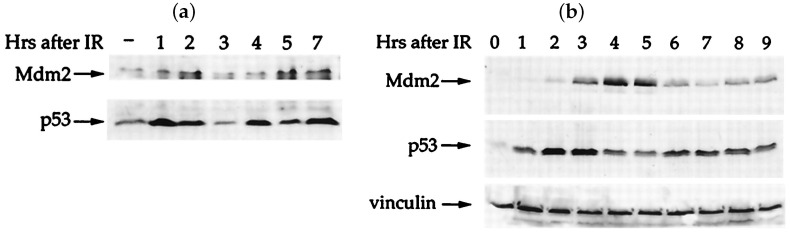
Example of damped oscillations in mouse fibroblasts NIH 3T3 cells (**a**) and human breast cancer MCF7 cells (**b**) following γ-irradiation when measured with Western blots, [18]. Copyright (2000) National Academy of Sciences, USA.

**Figure 2:**
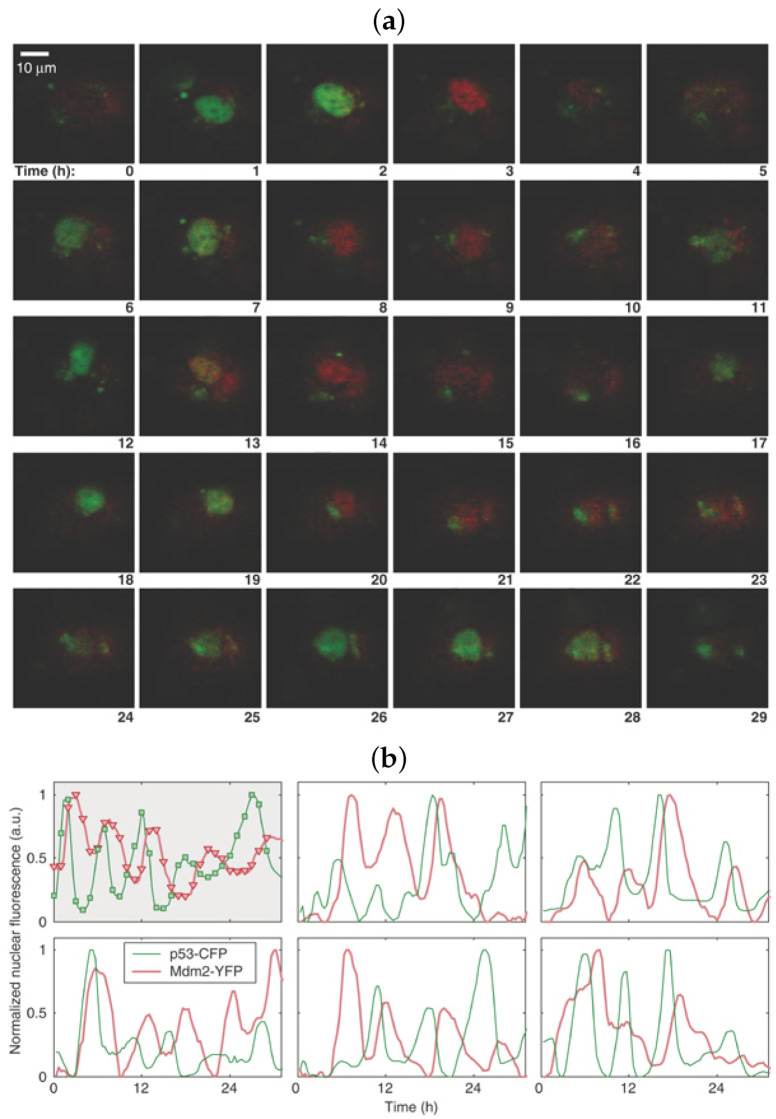
Examples of sustained oscillations in MCF7 cell following γ-irradiation obtained by time-lapse fluorescence microscopy, [19]. In (**b**), normalised fluorescence levels of p53 and Mdm2 from six different cells are shown, the top left plot in (**b**) shows oscillations in (**a**). Copyright (2006) reproduced with permission from John Wiley and Sons.

A common feature of many genetic pathways is periodic expression of proteins. In some instances this periodic expression is apparent and necessary at the cell population level, whereas for some genes, protein fluctuations may show variations between cells. The somatic clock gene, *Hes1*, is a good example for which oscillatory dynamics of its transcription factor protein exhibit at the population level. As part of the Notch signalling pathway, *Hes1* is a key gene involved in embryogenesis and relies on synchronous periodic protein expression across cells in order to form somites at regular temporal and spatial intervals. Fluctuating levels of Hes1 protein have been observed using immunoblots (western blots) [17]. With regards to p53, analysis of immunoblots at the cell population level have shown that p53 (and Mdm2) undergo damped oscillations after significant DNA damage and that the amplitude of oscillations is correlated to the amount of DNA damage—referred to as “analog” control, see Figure 1 [18]. However, in its role as a cellular gatekeeper, p53 levels will naturally vary from cell to cell, thus, while periodic fluctuations may be seen on the population level, the high inter-cellular variability means periodicities are perhaps more meaningful when observed for single cells. Lahav and coworkers have used time-lapse fluorescence microscopy to analyse p53 dynamics within individual cells [16,19,20]. They determined that sustained oscillations (with fixed amplitude and period) could be observed at the single cell level after γ-irradiation, see Figure 2, but that the number of periods (pulses) of p53 was directly correlated to the amount of DNA damage. They hypothesise that this “digital” control clock of pulses lasts until either a damage is repaired or the affected cell dies. Furthermore, they have shown how the source of the DNA damage (γ or UV radiation) can alter the dynamics exhibited by p53 (periodic or sustained expression) [15] and how such p53 dynamics affects the outcome for the cell (repair or senescence) [16]. We remark that p53 has been shown to oscillate in-vitro cancer cell cultures (MCF7—breast cancer, A549—lung cancer [21]), in-vitro healthy cells (RPE1—retinal epithelial [21]), as well as in-vivo [22].

**Figure 3:**
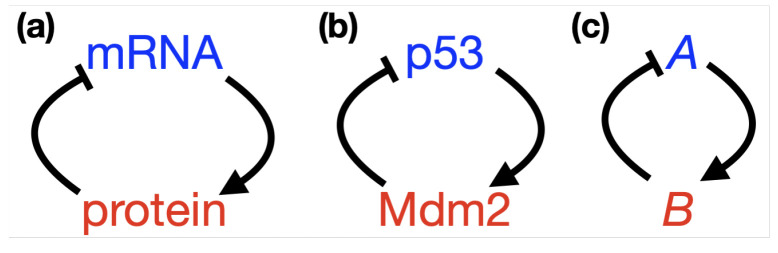
Schematics showing simple negative feedback loops between (**a**) Hes1 mRNA and protein, (**b**) p53 and Mdm2 and (**c**) *A* and *B*.

Evidently the dynamical behaviour of p53 is an important part of its function. There must be mechanisms within its pathway which drive both its periodic and sustained expression and account for the switch between these two states. Determining mechanisms which provoke oscillations in p53 GRN systems has been a major focus of mathematical endeavour. A common feature of GRNs, thought to hold the answer to oscillations, is negative feedback; both p53, and the aforementioned protein Hes1, are governed, at least in part, by negative feedback (see schematics in Figure 3). Hes1 offers a canonical example of a negative feedback loop in a GRN as a self-repressing gene; the translated gene protein binds with its own mRNA to inhibit future production, Figure 3a. For p53, the negative feedback is through interaction with (E3 ubiquitin-protein ligase) Mdm2. Mdm2 represses the transcriptional activity of p53 by binding to and ubiquitinating it, which marks it for degradation [23]. Mdm2 repression of p53 is important in processes such as wound healing, but if levels of Mdm2 are too high and repression is too strong can contribute to tumour formation, in this way Mdm2 is referred to as an oncogene [24]. To complete the negative feedback, activated p53 induces transcription of Mdm2 by binding to its promoter, Figure 3b [25]. Negative feedback intuitively lends itself as a candidate for oscillatory behaviour; considering Figure 3c, as the amount of *A* increases, binding and up-regulation of *B* becomes more likely, as the amount of *B* increases, binding and down-regulation of *A* becomes more likely and so on.

Early GRN modelling using Ordinary Differential Equations (ODEs) showed that the presence of negative feedback alone was insufficient to produce fluctuating protein levels [26,27,28]. Stability analysis has shown that an “intermediary” is required to push GRNs, with simple negative feedback, from regimes where they exhibit stable fixed points (sustained expression), into regimes where they give raise to a stable limit cycle and exhibit periodic fluctuations. The intermediary may be the time taken for molecular processes to occur (time delays), the requirement for molecules to move to certain spatial locations (spatial effects) or additional/intermediary interactions with other molecules (e.g., positive feedback). Mathematical models of GRNs take into account some or all of these processes. Time delays have been incorporated into models of Hes1 and other known genetic pathways [29,30,31,32,33,34,35,36,37,38], as well as being incorporated into generic GRN models [39,40,41,42,43,44,45,46,47,48,49] in order to provoke oscillations. Models coupling positive feedback with negative feedback have also had success in deriving oscillatory dynamics [50,51,52,53,54,55,56]. Diffusion of genetic molecules as well as compartment models which take into account the fact that different processes occur at different locations within the cell have been investigated [57,58,59,60,61,62,63,64,65,66]. For an excellent discussion of the key mathematical components which permit biochemical oscillations see either the review [67] or book [68].

Beyond the fact that negative feedback alone is insufficient to provoke stable oscillations, the simple two-gene GRN between p53 and Mdm2 is a simplification of the highly complex genetic pathway within which they act. This review will focus on mathematical modelling of p53. In Section 2 we will consider efforts to induce oscillatory dynamics by incorporating further aspects of the p53 genetic pathway, both implicitly, by incorporating additional genes and additional feedback loops (both positive and negative), and explicitly by including time delays and diffusion. In Section 3 we will compliment this with a discussion of how biology has informed and validated mathematical models of p53 and how mathematical modelling offers feedback to experimentalists, posing additional questions which inform experiment design. In Section 4 we pose questions to the community—where do we go from here? We hope the review will provide a simple reference guide as to the different ways in which mathematical models can be deployed to model p53, as well as showcasing the state-of-the-art of this vibrant research field.

## 2. Mathematical Modelling of p53

In order to facilitate the following discussion, we commence by providing details of how the negative feedback network of p53 and Mdm2, Figure 3b can be modelled, and show that such a model fails to support oscillatory dynamics. In the following information box the model is described in brief, with corresponding equations. These equations are solved and simulated to show the dynamic behaviour of both p53 and Mdm2. All future subsections of this Section will display similar boxes in order that the reader can easily compare and contrast the different modelling approaches. The simulation of the different equation systems was carried out using Matlab except for the spatial model which was numerically solved using FreeFem++ software, the Matlab and FreeFem++ scripts have been made available as part of this publication [69].

- p53-Mdm2 negative feedback loop

Although there are several ways in which the negative feedback network between p53 and Mdm2 depicted in Figure 3b can be translated into mathematical equations and modelled, we have chosen a simple pair of rate equations for the concentration of p53 (denoted by *x*) and the concentration of Mdm2 (denoted by *y*). Namely, we consider the following system of Ordinary Differential Equations (ODEs):

(1) $$\begin{array}{cc} \dot{x}\left(t\right) & =k_{s}-k_{1}y\left(t\right)\frac{x(t)}{K_{1}+x\left(t\right)}-d_{x}x\left(t\right),\\ \dot{y}\left(t\right) & =k_{2}\frac{x{\left(t\right)}^{n}}{K_{2}^{n}+x{\left(t\right)}^{n}}-d_{y}y\left(t\right). \end{array}$$

**Figure 4:**
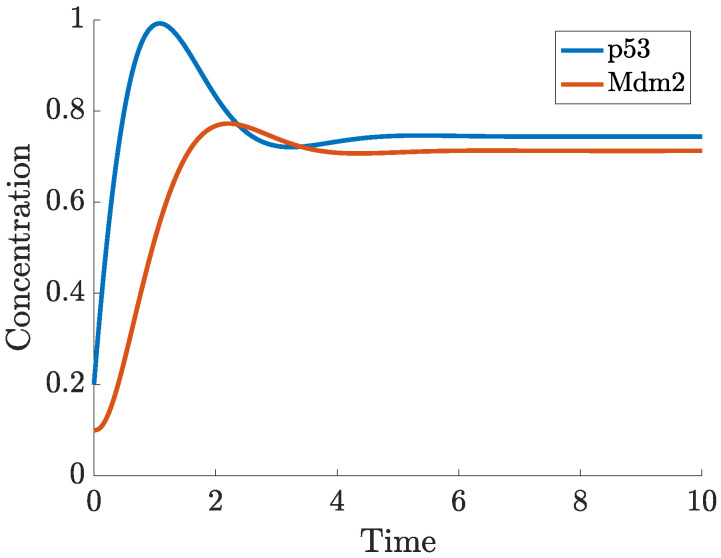
Solution to (1) starting from (0.2,0.1) at t=0.

**Figure 5:**
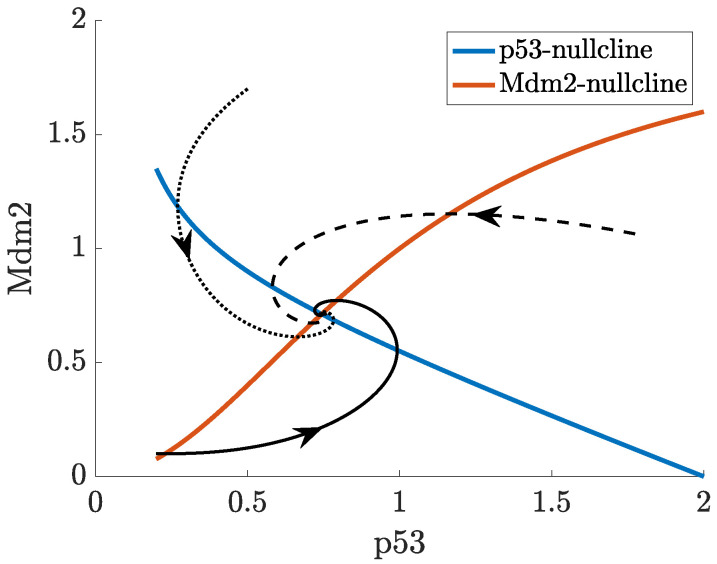
Three trajectories (the solid one is from Figure 4) and nullclines in the p53-Mdm2 phase plane.

In (1), p53 is produced at a constant rate, $k_{s}$, and degraded either in an Mdm2-dependent manner (modelled by a Michaelis-Menten function), or independently of Mdm2 with the rate of degradation, $d_{x}$. Mdm2 is produced at a rate which depends on the concentration of p53 (modelled by a Hill function with coefficient *n*) and degraded at a rate $d_{y}$. All solution trajectories (x,y) of (1), regardless of initial conditions, converge eventually to a single positive steady state (fixed point), which is determined by the model parameters (given in Appendix A) and located where the curves $\dot{x}\left(t\right)=\dot{y}\left(t\right)=0$ intersect (these curves are called the nullclines of the system). Figure 4 shows a single trajectory from an initial state, (0.2,0.1), towards the stable state (0.74,0.71). The Mdm2-p53 phase plane in Figure 5 displays the dynamics of (1) from a different perspective. In this plot, the nullclines (expressed as functions, y(x)) intersect at one point towards which all trajectories of the system tend. Three such trajectories from non-trivial initial states are shown in black; the solid trajectory curve corresponding to the solution in Figure 4. We refer to the book of Hirsch, Smale and Devaney [70] for a suitable introduction into dynamical systems such as system (1) and global nonlinear techniques, for example, using nullclines to analyse such systems.

### 2.1. Time Delays in p53 Modelling

In the simplest negative feedback model of the p53-Mdm2 pathway, Figure 3b, an assumption is made that both p53-mediated transcription of Mdm2 and Mdm2-mediated ubiquitination of p53 are instantaneous and occur on the same time-scale. One class of alterations to the simple negative feedback loop is to incorporate a time delay (or indeed multiple). Consider the upregulating arm of Figure 3b; as a transcription factor protein, p53 binds to the promoter of Mdm2, which synthesises Mdm2 mRNA, which in turn translates into Mdm2 protein. Inherently, these biological processes take a certain amount of time; time for protein-promoter binding, transcription and protein synthesis [71,72,73]. On the other hand while ubiquitination of p53 is a rapid process, it is still not instantaneous.

**Figure 6:**
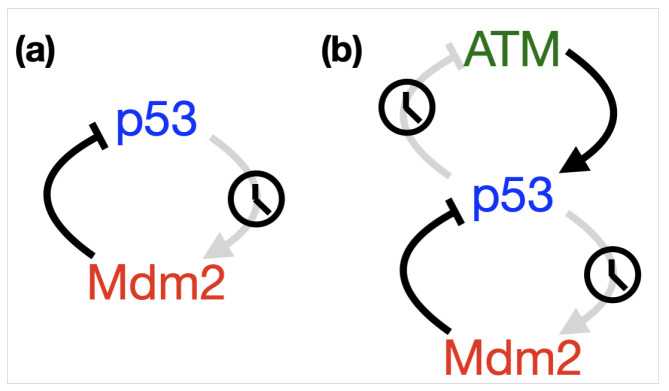
Schematics showing where time delays have typically been incorporated into (**a**) the simple negative feedback between p53 and Mdm2 and (**b**) the simple p53-Mdm2 negative feedback loop coupled with the negative feedback p53-Wip1-ATM pathway.

Numerous authors have focussed on incorporating delays into their models of the p53-Mdm2 pathway. This can be traced back to Lev Bar-Or et al., who used an alternative although equivalent approach to account for a delay in the system, by introducing an hypothetical intermediary in the positive arm of the feedback loop. Matching to experiments they were able to show that a “delay” is an essential ingredient in order to provoke oscillations [18]. Although such an intermediary has not been discovered, their conclusions have been confirmed by authors incorporating an explicit time delay [31,74,75,76,77,78,79]. In Figure 6a we indicate how the simple p53-Mdm2 negative feedback loop is altered by incorporating such a delay; a single delay (represented by the clock symbol) accounts for any of the processes involved in upregulation of Mdm2 protein from p53. Monk suggests that transcriptional delays are of the order of 10–20 min, while translational delays are of the order of 1–3 min, so transcriptional delays dominate the process and all other delays can be absorbed into a single delay. By incorporating a single transcriptional delay of 15 min they were able to derive robust oscillations in the p53-Mdm2 system with a period of 3 h [31], matching those seen in experimental observations [18]. Tiana et al. showed that delay is an essential ingredient of the p53-Mdm2 system [75], in order to capture the experimentally observed dynamics of p53 and Mdm2 following DNA damage [23]. Yan & Zhuo show that significant oscillations can follow DNA damage providing a delay in the range τ≈20–60 min is incorporated into their negative feedback model. Botanni & Grammaticos show that a delayed negative feedback model can switch between a sustained low expression of p53 and oscillations depending on whether stress/damage has occurred; their findings indicate that the stability of the non-stressed system depends on basal and degradation levels of Mdm2 [78]. In these models the delay typically acts as a bifurcation parameter, which exhibits a Hopf bifurcation; such a bifurcation occurs at the point of transition from existence of a stable fixed point (sustained expression), to existence of a limit cycle (oscillatory expression) [79,80]. Ogunnaike takes a systems engineering approach to show how a minimal negative feedback model with delay can account for the apparent dichotomy between experimental results for single cell lines and cell populations, showing how “digital” behaviour at the single cell level presents as “analog” behaviour at the cell population level [76]. In the following information box we outline a simple delayed negative feedback model corresponding to Figure 6a and show how the incorporation of a single delay can push the system from non-oscillating to oscillating.

- p53-Mdm2 negative feedback loop with delay

Due to a delay in the expression of Mdm2, as depicted in Figure 6a, we can assume that the Mdm2-dependent degradation of p53 depends on the concentration of Mdm2 at some previous time. Indeed, not all Mdm2 molecules that exist at the time *t* can target nuclear p53 for degradation, for example, because some molecules are still localised at the site of mRNA translation in the cytoplasm. We can modify the simple negative feedback system (1) by introducing a delay parameter τ such that the concentrations of p53 and Mdm2 follow a system of Delay Differential Equations (DDEs):

(2) $$\begin{array}{cc} \dot{x}\left(t\right) & =k_{s}-k_{1}y\left(t-\tau \right)\frac{x(t)}{K_{1}+x\left(t\right)}-d_{x}x\left(t\right),\\ \dot{y}\left(t\right) & =k_{2}\frac{x{\left(t\right)}^{n}}{K_{2}^{n}+x{\left(t\right)}^{n}}-d_{y}y\left(t\right). \end{array}$$

**Figure 7:**
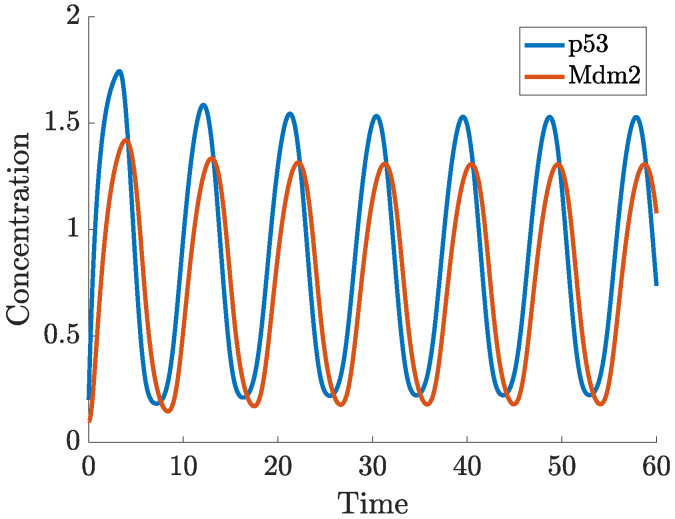
Solution to (2) starting from (0.2,0.1) at t=0.

**Figure 8:**
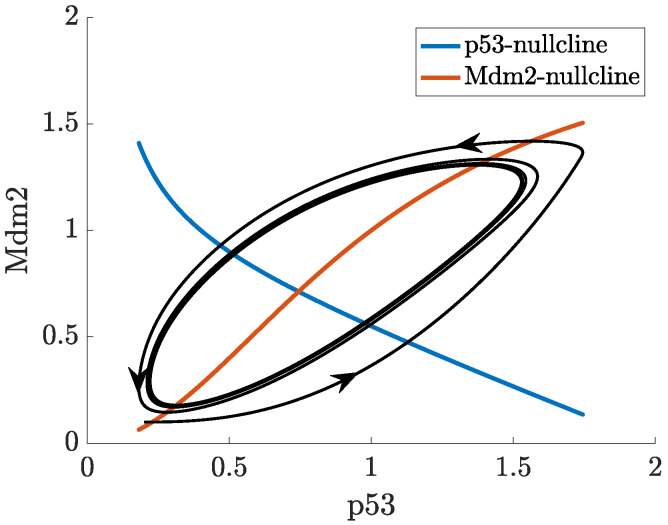
Trajectory from Figure 7, limit cycle and nullclines in the p53-Mdm2 phase plane.

**Figure A1:**
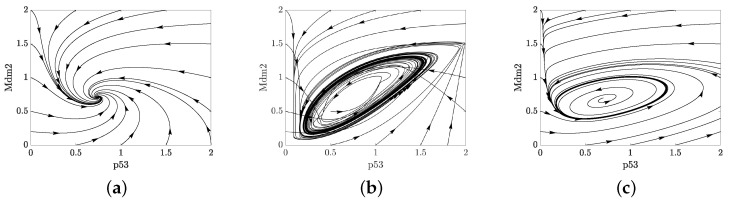
(**a**) Vector-field for model (1). (**b**) Vector-field for model (2). (**c**) Vector-field for model (4). Vector fields for the systems of Equations (1), (2) and (4), given in Section 2 corresponding to a negative feedback model, time delayed model and coupled positive and negative feedback model, respectively. These vector fields can be thought of as representing a flow in a state space given by the differential equations. Roughly speaking, wherever we position the concentrations of p53 and Mdm2 in the p53-Mdm2 planes as above, these concentrations are either “flowing” down to a stable steady-state (**a**) or spiralling towards a stable limit cycle in a way as shown in the plots (**b**,**c**). In (**b**,**c**) we always draw one trajectory that spirals out from inside of the limit cycle.

In (2), the actual concentration of Mdm2, which regulates p53 at the time *t* is taken as the concentration of Mdm2 at the time t−τ. In this case, oscillations in the concentration of p53 and Mdm2 may appear for a sufficiently long delay, as can be seen in Figure 7. While the nullclines of the DDE system (2) do not differ from the nullclines of (1), the (x,y) trajectory from Figure 7 settles into a sustained oscillatory path, called a limit cycle, shown in the phase-plane in Figure 8. Depending on the initial state, some solutions to (2) may show oscillations with varying amplitudes and period before they finally approach the limit cycle, this is shown in the vector field plot given in Figure A1b in Appendix A.

More complex models including other genes/feedback loops have also been investigated incorporating delays. Mihalas et al. developed their earlier single delay model [74] by introducing three delays, one in each of the positive and negative arm of the negative feedback loop, and an additional delayed interaction with HAUSP (herpesvirus-associated ubiquitin-specific protease), which downregulates Mdm2 by deubiquination of both p53 and Mdm2 [81]. By simplifying this model so that all three delays are equivalent, and using this single delay parameter as a bifurcation parameter, they show that different parameter regimes can lead to different dynamic behaviours: sustained expression, damped and non-damped oscillations of p53.

Lahav and coworkers have a long history of investigating the p53-Mdm2 pathway dynamics; providing an interdisciplinary approach by performing both experiments as well as analysis of mathematical models. Geva-Zatorsky et al. considered several different network models spanning from the basic negative feedback loop model, to models including positive feedback (see Section 2.3) or a time delay. They were the first to introduce what they called a checkpoint inhibitor model which consisted of two coupled negative feedback loops; the simple p53-Mdm2 delayed negative feedback loop coupled to a longer negative feedback loop in which a protein downstream of p53 inhibits an upstream signalling protein for p53. Although they didn’t specify the specific proteins which formed the second negative feedback loop, this network motif was found to be particular robust to generating sustained oscillations for a wide range of parameters [19]. Batchelor et al. specifically looked for protein candidates which fit this check point inhibitor model, and by using mathematical modelling in synergy with experiments were able to suggest that good candidates for these additional proteins are ATM (ataxia telangiectasia mutated serine/threonine kinase) and Wip1 (wild-type p53-induced phosphatase 1) [82]. ATM, triggered by DNA damage, phosphorylates and activates p53, while it is concurrently downregulated by Wip1 an additional target protein for the transcription factor p53. Purvis et al. used such a model to investigate the role p53 dynamics (pulsed or sustained expression) has on cell fate, and whether drug treatments could alter the dynamics and hence the outcome for the cell [16]. In Figure 6b we indicate how the negative p53-Mdm2 feedback loop is coupled to a negative feedback loop between p53 and ATM. In this case the delay (clock symbol) on the negative arm of the loop, accounts for the time taken for p53 to transcribe Wip1 and Wip1 to downregulate ATM; Wip1 being modelled either implicitly or explicitly depending on the model. In recent years, a common extension when investigating the role of delays is to consider the extended model given in Figure 6b. It has been shown that the delays in both negative feedback loops are important components to derive oscillatory behaviour [83,84]. One difficulty though is that in order to make analysis of the models tractable it has, so far, required that both delays are assumed to be equal, which may or may not be the case in the real biological system.

Before concluding our section on time-delays, in models of p53 it is important to discuss models which use distributed rather than fixed delays [85,86,87]. In their conclusions, Monk reminds the author that to be biologically accurate delays should be taken from a distribution rather than having a discrete value [31]. Neamtu and coauthors have shown that oscillatory dynamics occur for a model of p53-Mdm2 with continuous delay kernels [86,87]. It would appear, however, that no additional gain (for example, dynamics which more closely resemble the experimental results) is derived from using such distributed delay models, and their increase in complexity leads to a loss in tractability, thus, authors tend to favour incorporating the simpler discrete time delay.

### 2.2. Incorporating Diffusion in p53 Modelling

**Figure 9:**
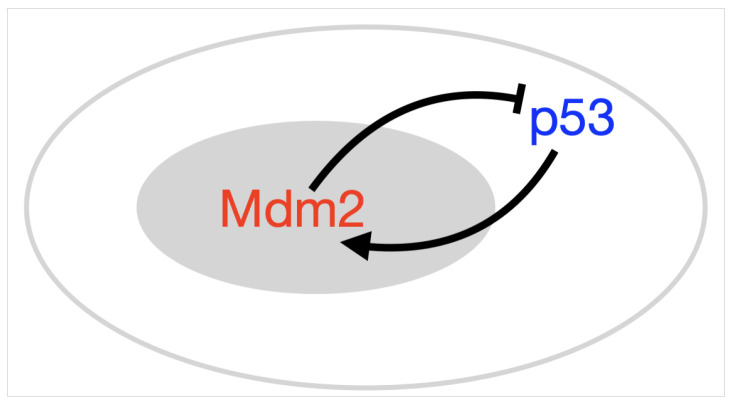
Schematic showing the simple negative feedback between p53 and Mdm2 overlayed on an oval cell with central shaded nucleus indicating that different processes occur at different locations within the cell.

Rather than use explicit time delays to account for the intermediary processes which occur in the p53 genetic pathway, some authors model the time taken for processes to happen intrinsically by considering space. The effects of space may be taken into account explicitly, using reaction-diffusion Partial Differential Equations (PDEs), and/or implicitly, using compartment model approaches. In eukaryotic cells mRNA is transcribed in the nucleus and protein is translated in the cytoplasm. A key motivation behind spatial models is accounting for the mRNA transport between these two compartments. In Figure 9 we provide a simplified schematic showing how the p53-Mdm2 negative feedback system is incorporated into a simple spatial model; p53 translated in the cytoplasm (white space) must bind with the Mdm2 promoter in the nucleus (shaded region) to transcribe it. Note, that although an oval shape with off-centred nucleus is depicted spatial models have accounted for a variety of different cell shapes.

An early spatial model was investigated by Gordon et al. [88]; they investigated a distributed delay p53-Mdm2 model but also included diffusion of molecules, converting to a PDE rather than ODE approach. In order to account for different processes occurring at different parts of the cell, they also incorporated two compartments (the nucleus and cytoplasm) of the cell and added weightings to the rates of protein synthesis in which protein is only synthesised in the cytoplasm, the weighting decreasing with distance from the nucleus. They focussed their investigation on the affect the shape and size of the cellular domain (as well as the position of the nucleus within it) has on oscillations, predicting that oscillations with high amplitude are more common in circular cellular domains with a central nucleus. Their results indicated that p53 oscillations could alternate between high and low amplitude, and argue that the digital pulses captured in experimental results are only a snapshot of the full dynamic behaviour. Spatial models of p53 can capture oscillatory behaviour even without delays, Sturrock et al. were the first authors to show that a purely spatial model of p53 could lead to oscillatory dynamics [89]. Their compartmental PDE model was able to capture temporal oscillations, which match those observed experimentally, but they also show that the additional spatial oscillations agree with those seen in [19]. Their model predicts an optimum distance from the nucleus for protein synthesis in order to derive sustained high-amplitude oscillations [89], a typical result of spatial models [64]. The authors extended their model to include active transport (by way of a convection term) across the nuclear membrane and carry out their analysis on a cellular domain derived from an image of an osteosarcoma cell [90]. They find that such a model is more robust to oscillatory dynamics, and furthermore, that active transport alone in the absence of diffusion can still lead to oscillations. Dimitrio et al. confirm that the introduction of space is sufficient to push the p53-Mdm2 system from non-oscillatory to oscillatory dynamics [91]. They first consider a simple compartment model; the authors include ATM mediated damage as an activator of the p53-Mdm2 system, but rather than include it as an additional molecule with its positive pathway (see Section 2.3) they simply incorporate it as an additional parameter, the size of which correlating to the amount of damage. This parameter is a bifurcation parameter; for values of ATM within a fixed range the system exhibits oscillations. They extend their model to a PDE system including diffusion; again ATM is a bifurcation parameter but they show that dynamics are governed by various spatial aspects including diffusion rates, the permeability of proteins and cell volume. In the following information box we create a simple spatial model for p53-Mdm2, which introduces diffusion of the gene species, as well as segregating processes into nuclear and cytoplasmic compartments as per Figure 9; oscillatory dynamics are derived.

- p53-Mdm2 negative feedback loop with diffusion

The physiological delay in the expression of Mdm2 can be achieved by introducing a spatial element into models, for example, by considering single cells with two compartments (nucleus and cytoplasm), and by differentiating between the processes which happen exclusively in the nucleus (e.g., gene transcription), from those which happen in the cytoplasm (e.g., translation), see Figure 9. Thus, we may extend the traditional negative feedback model, by assuming that the concentrations of p53 and Mdm2 depend, not only on time, but also on space. Moreover, we introduce, an additional gene product, Mdm2 mRNA (denoted by *z*), and assume that p53-dependent transcription of Mdm2 gene into mRNA occurs in the nucleus (in the simulation below we specified a small area inside the nucleus, the so-called DNA locus, where the gene of Mdm2 is localised), whilst the translation of mRNA into the protein occurs in the cytoplasm (in the simulation below we assumed that ribosomes are localised in a small annulus at some short distance from the nuclear membrane). Hence, we introduce the following system of Partial Differential Equations (PDEs), in which the subscripts, *n* and *c*, denote, respectively, the nuclear and cytoplasmic concentrations of p53, Mdm2 and its mRNA:

(3) $$\begin{array}{cccc} \frac{dx_{n}}{dt} & =-k_{1}y_{n}\frac{x_{n}}{K_{1}+x_{n}}-d_{x}x_{n}+D_{x}\Delta x_{n}, & \frac{dx_{c}}{dt} & =k_{s}-k_{1}y_{c}\frac{x_{c}}{K_{1}+x_{c}}-d_{x}x_{c}+D_{x}\Delta x_{c},\\ \frac{dy_{n}}{dt} & =-d_{y}y_{n}+D_{y}\Delta y_{n}, & \frac{dy_{c}}{dt} & =k_{tra}z_{c}-d_{y}y_{c}+D_{y}\Delta y_{c},\\ \frac{dz_{n}}{dt} & =k_{2}\frac{x_{n}^{n}}{K_{2}^{n}+x_{n}^{n}}-d_{z}z_{n}+D_{z}\Delta z_{n}, & \frac{dz_{c}}{dt} & =-d_{z}z_{c}+D_{z}\Delta z_{c}. \end{array}$$

**Figure 10:**
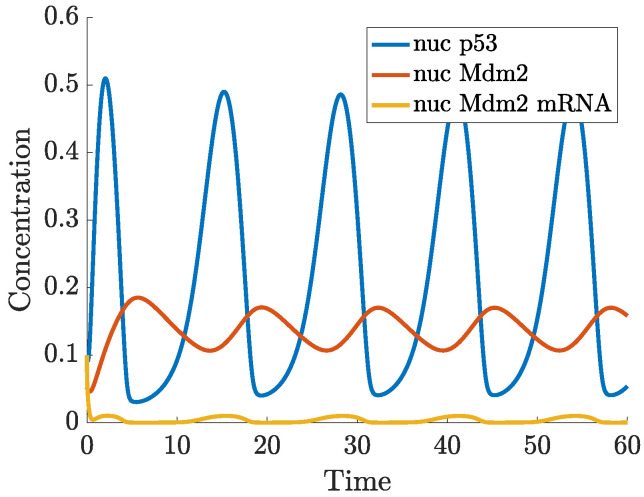
Total nuclear concentration of p53, Mdm2 and Mdm2 mRNA.

**Figure 11:**
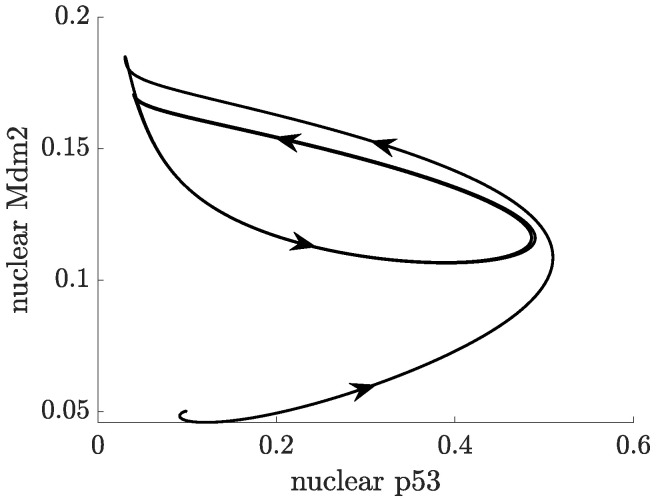
Trajectory from Figure 10 and limit cycle in the Nuclear p53-Nuclear Mdm2 phase plane.

**Figure 12:**
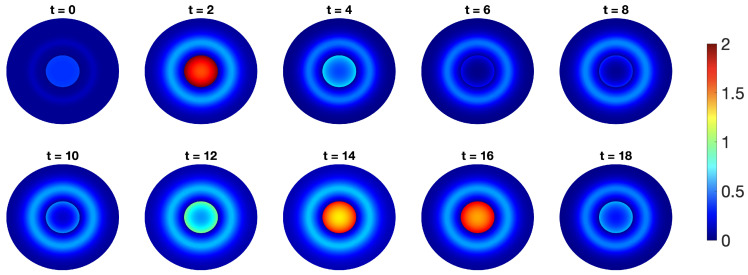
2-D visualisation of p53 concentration.

In (3), Mdm2-dependent degradation of p53 happens in both the nucleus and cytoplasm. The p53-dependent synthesis of Mdm2 is now a two-step process: first Mdm2 mRNA is produced in the nucleus (modelled by a Hill function), and then mRNA is translated into Mdm2 in the cytoplasm, with a rate of translation $k_{tra}$. p53 is produced in the cytoplasm, at a constant rate $k_{s}$. Natural degradation is denoted by $d_{x}$, $d_{y}$ and $d_{z}$. All three species are assumed to diffuse freely and randomly in the cell (modelled by a Laplacian Δ), with the diffusion rates denoted by $D_{x}$, $D_{y}$ and $D_{z}$. In the simulation below, it is assumed that mRNA can shuttle from the nucleus to the cytoplasm, whereas p53 and Mdm2 proteins migrate from the cytoplasm to the nucleus only. Transport through the nuclear membrane is assumed to be passive and given by a single parameter called membrane permeability. Oscillatory solution trajectories are confirmed in both the dynamic plot in Figure 10, and by the presence of a limit cycle in the phase-plane in Figure 11. Furthermore, oscillations of p53 can be seen the 2D spatio-temporal plots, depicted in Figure 12. Parameters are provided in Appendix A. Unlike in Equations (1) and (2), the time derivative is denoted by d/dt in (3), the commonly used notation in PDEs.

Eliaš and coauthors expand the previous compartmental and spatial model of Dimitrio et al. by including equations for ATM and Wip1 as sufficient and necessary elements in the p53 oscillatory response to DNA damage, as per [82]. In [92], a compartmental ODE model for the intracellular dynamics of p53, with ATM and Wip1 as essential parts of the DNA damage signalling pathway, is proposed. In this model, ATM dimer dissociation and activation is due to a DNA strand breaks sensor, *E*, which can be either the Mre11/Rad50/Nbs1 complex [93], or a hypothetical molecule produced by changes in chromatin structures after DNA damage [94]. Bifurcation analysis with respect to the parameter *E*, which is assumed to be positively correlated with the extent of DNA damage, reveal the existence of two critical values; for values of *E* between these two critical values, p53 and other species oscillate sustainedly. If the DNA damage signal is too weak, oscillations are not found at all; if the damage signal is too high, only damped oscillations, as in [19], appear and the number of pulses decreases with the increasing value of *E*.

**Figure A2:**
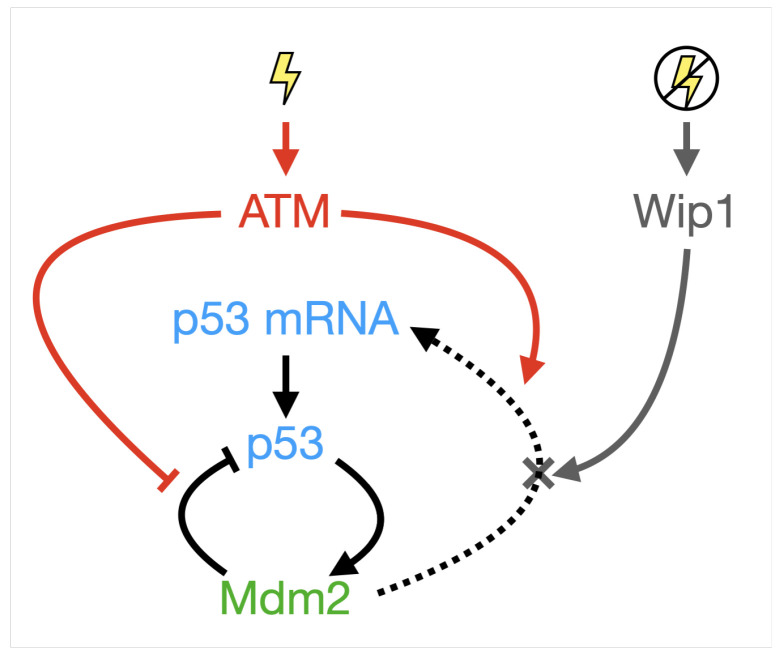
A simplified schematic indicating the novel model presented in which Mdm2 can act as either a promoter or inhibitor of p53 depending on the damage signal and mediated by ATM and Wip1. Specifically, when the damage signal is present ATM and the red pathway is activated in which ATM mediated Mdm2-p53 mRNA binding promotes p53 synthesis and inhibits Mdm2 p53 protein degradation; when the damage signal is removed the red pathway is turned off and in addition the grey pathway mediated by Wip1 is turned off which prevents Mdm2-mediated p53 synthesis through Mdm2-p53 mRNA binding.

Spatial models similar to those of Sturrock et al. [89] and Dimitrio et al. [91], but including ATM and Wip1 signalling, were proposed in [95,96,97]. The models in [92,95,96] are built on an observation that stabilisation of p53 in the nucleus follows from the previous phosphorylation of p53 by ATM [11]. The spatial oscillator in [97] relies on a dual positive and negative regulation of p53 by Mdm2 as suggested by Gajjar et al. [98]. Indeed, experiments of Gajjar and collaborators suggest that ATM-mediated phosphorylation of p53 may neutralise the negative effect of Mdm2, rather than actively up-regulating p53. ATM-dependent phosphorylation of Mdm2 is shown to lead to the binding of phosphorylated Mdm2 to p53 mRNA. Enhanced synthesis of p53 from such mRNA-Mdm2 complexes, stimulates p53 activity and suppresses Mdm2-dependent degradation of p53. Once Mdm2 phosphorylation is reversed, by Wip1, Mdm2 switches to become a negative regulator of p53 activity, by promoting p53 polyubiquitination and degradation [98]. This dual role of Mdm2 towards p53 was shown to yield oscillations of p53 in [97]. Moreover, this mechanism can even explain spontaneous pulses of p53, as observed in [15,99]. Simulations in [97] suggest that even short time signalling, via ATM (∼1 h), may be sufficient to create a pool of p53 mRNA-Mdm2 complexes, which serve as a reservoir for stable copies of p53, long after the ATM signalling is turned off, permitting 6 h long pulses of p53. In Appendix B we present a schematic of a novel model, Figure A2, which takes into account the dual response of Mdm2 mediated by ATM and Wip1. While not strictly within the remit of this review, this new model based on the mechanisms proposed by Gajjar et al. may form the basis of future work by the authors.

### 2.3. Positive Feedback in p53 Modelling

**Figure 13:**
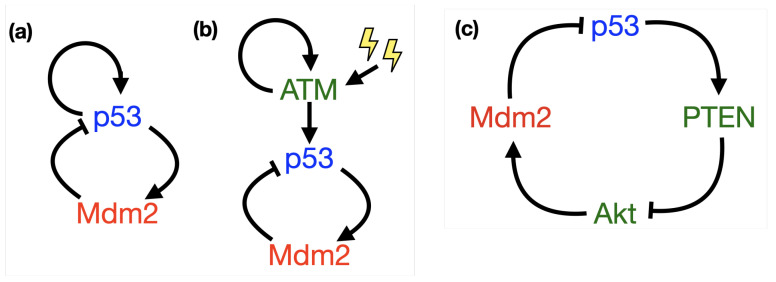
Schematics showing positive feedback loops involving p53-Mdm2: the simple negative feedback between p53 and Mdm2 is coupled to (**a**) a positive undetermined feedback on p53 or (**b**) a positive feedback loop of autocatalytic ATM. Panel (**c**): a positive feedback loop of p53-PTEN-Akt-Mdm2 which contains two inhibitory arms which combine to make this pathway “globally” positive.

An alternative approach when developing a p53-Mdm2 network model is to incorporate additional steps or intermediaries, which add additional upregulating pathways. The simplest way in which to couple a positive feedback loop to the negative p53-Mdm2 system is to include a positive feedback loop on p53, a type of model coined as a relaxation oscillator by [19]. This simple positive feedback loop, depicted in Figure 13a, could account for any number of additional p53 pathway components which in total have an upregulating effect on p53, but may also account for autocatalysis of p53. Ciliberto et al. consider an additional mutual inhibition between p53 and Mdm2 as their positive feedback [100]. They take advantage of incorporating both positive feedback and spatial aspects by using a compartment model, in which they segregate processes and gene products by location (cytoplasm or nucleus); their results quantitively and qualitatively match the experimental results of [20]. They conclude that the positive feedback creates a bistable system of both high and low levels of p53, but the negative feedback breaks this stability causing oscillations between the high and low states. In the following information box we create a simple model for p53-Mdm2, which couples the typical negative feedback with auto-catalytic positive feedback, as in Figure 13a; oscillatory dynamics can be derived.

- p53-Mdm2 negative feedback loop with a positive feedback

If we assume that a physiological delay in the p53-Mdm2 auto-regulatory network is attained through auto-catalytic positive feedback, as shown in Figure 13a, then such upregulation of p53, perhaps due to p53 downstream target(s), can be effectively modelled with a sufficiently nonlinear increasing function of the p53 concentration. By introducing a second Hill function, as a positive auto-catalytic feedback term for p53, we obtain a new system of ODEs:

(4) $$\begin{array}{cc} \dot{x}\left(t\right) & =k_{s}+k_{p}\frac{x{\left(t\right)}^{m}}{K_{p}^{m}+x{\left(t\right)}^{m}}-k_{1}y\left(t\right)\frac{x(t)}{K_{1}+x\left(t\right)}-d_{x}x\left(t\right),\\ \dot{y}\left(t\right) & =k_{2}\frac{x{\left(t\right)}^{n}}{K_{2}^{n}+x{\left(t\right)}^{n}}-d_{y}y\left(t\right). \end{array}$$

**Figure 14:**
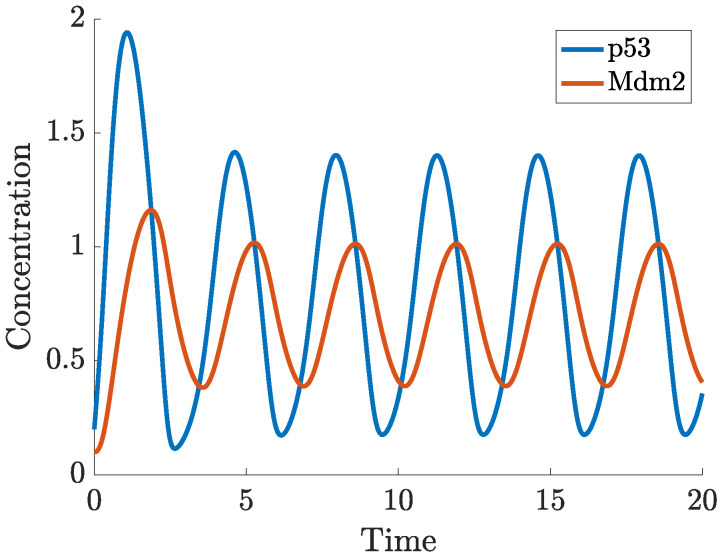
Solution to (4) starting from (0.2,0.1) at t=0.

**Figure 15:**
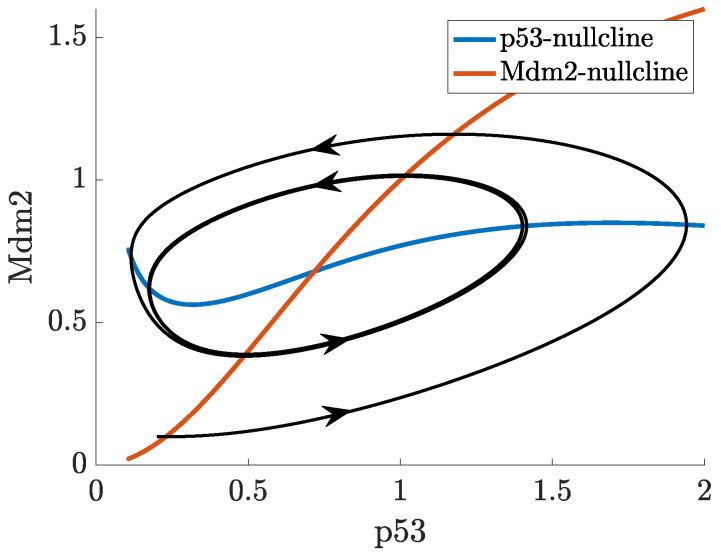
Trajectory from Figure 14, limit cycle and nullclines in the p53-Mdm2 phase plane.

This system yields an oscillatory response, for a suitable choice of parameters (given in Appendix A). In Figure 14 we show the time-dependent solution of the system, for given initial conditions, which exhibits clear periodic fluctuations. In Figure 15, the phase-plane plot shows both the nullclines of the system and the solution trajectory corresponding to Figure 14, which tends to a limit cycle. The vector field is provided in Figure A1c in Appendix A.

Zhang et al. study, and compare four different positive feedback mechanisms incorporated into the negative p53-Mdm2 system: Mdm2 as a promoter as well as an antagonist of p53; autocatalytic p53; autocatalytic Mdm2; p53 as a downregulator of Mdm2 [101]. Recall, that changes in dynamics of p53 from sustained expression to periodic expression are governed by bifurcations. We have previously mentioned Hopf bifurcations, such bifurcations are described as either supercritical or subcritical, depending on whether they give raise to a stable or unstable limit cycle; many models give rise to supercritical Hopf bifurcations. Zhang et al. find that their models give rise to either a subcritical Hopf bifurcation, or a different kind of bifurcation called a saddle-node bifurcation (on an invariant circle) and discuss that these bifurcation types lead to more robust high amplitude oscillations, compared to those for supercritical Hopf bifurcation models, although they do conclude that supercritical Hopf bifurcation dynamics more closely fit the experimental results of [19]. For more detailed discussion of bifurcation types and behaviours in GRNs we direct the reader to [80].

Other models have incorporated a positive feedback loop on ATM (in this case without Wip1) [102,103,104,105]. Frequently, such models treat ATM as a switch, which responds to DNA stress (typically double stranded breaks caused by γ-irradiation); specifically ATM is “on” when damage occurs, and as damage is repaired and the signal reduces, ATM is turned “off”. ATM is known to be autocatalytic, or self-generating, and so creates positive feedback. In Figure 13b we indicate how the negative p53-Mdm2 feedback loop is coupled to the positive feedback of self-promoting ATM, which is activated by irradiation-induced damage (lightning symbols). Ma et al., like Ciliberto et al. [100], use a compartment model, but use the ATM switch as the positive feedback loop, rather than a positive feedback loop on p53. They show that they can recreate the “digital” response of single cells to radiation, in which an increase in radiation dose leads to an increase in number of undamped oscillations; they argue that net response at the cell population level would manifest as damped oscillations [102]. Jun-Feng & Ya also reproduce the “digital” behaviour for the ATM switch, but under a delayed, rather than a compartment model, finding also that the period of oscillations increases with increase to the delay parameter [105]. Wagner et al. use a delayed negative feedback model, rather than a compartment model, and incorporate ATM as a continuum, rather than as being on/off. They argue that while the delay in the negative feedback loop leads to oscillations, the ATM parameter also acts as a bifurcation parameter, and can stabilise oscillations by both enhancing the system feedback and decreasing dampening [103]. Chickarmane et al. incorporate two positive feedback loops into their model, both autocatalysis of p53 and the ATM switch, essentially a hybrid of Figure 13a,b. Their stochastic model is found to give rise to robust oscillatory dynamics [104].

Another frequent approach is to couple the p53-Mdm2 negative feedback loop to the more complex p53-PTEN-Akt-Mdm2 pathway, which is “globally” positive, see Figure 13c. By “globally” positive we mean that the feedback loop contains an even number of inhibitory/negative arms, which effectively sum to being positive. This is essentially a model of the type depicted in Figure 13a, but where the positive pathway has been explicitly described. Phosphatase and tensin homolog (PTEN), like p53, is a tumour suppressor and another gene commonly mutated in humans cancers, it is trans-activated by p53. Protein kinase B (PKB), otherwise known as Akt, provides an additional connection from p53 to Mdm2; Akt is repressed by PTEN (by dephosphorylation) but activates Mdm2 (by phosphorylation). Wee and Aguda were one of the first to model this second pathway [106]. In fact, their model takes mutual inhibitory network, between p53 and oncogenic Akt, as a starting point, upon which they add interactions with Mdm2 and PTEN (via PIP3, phosphatidyl inositol-3,4,5-trisphosphate); their interest being directed towards showing that the system exhibits a robust bistable switch controlling cell fates: survival or death, rather than looking for oscillatory dynamics. Dai et al. consider the coupled system of the negative p53-Mdm2 pathway, with the positive p53-PTEN-Akt-Mdm2 pathway; they incorporate two delays (the transciptional delay discussed above from p53 to Mdm2 as well as a delay between p53 and PTEN) and rather than considering the system under stress, they consider it driven by a growth factor which activates both Atk and Mdm2. Their results indicate that delays are still critical to deriving oscillatory behaviour, with the p53-Mdm2 delay remaining a key bifurcation parameter, causing a Hopf bifurcation, as well as showing how changes to the length of the delays can change the nature of the oscillations [107]. Puszynski and coworkers also consider the addition of the positive PTEN-Akt pathway; their complex model, in some ways can be seen as a hybrid of all of the modelling approaches reviewed here, as they incorporate a physiological delay in the form of additional gene products/intermediaries and compartmentalise the model into nucleus and cytoplasm, as well as coupling the negative feedback with positive feedback [108]. Their model suggests that the presence of the positive feedback loop actually acts to suppress long-lasting oscillations after DNA damage, imposing a clock for damage repair beyond which the cell will favour apoptosis instead.

## 3. Interplay between Experiments and Modelling

### 3.1. It All Started with Galit Lahav

As discussed in Section 1, negative feedback lies at the root of every known oscillating system, since it brings a GRN back to the “starting point” of its oscillation [67]. However, a two-step negative feedback loop, as depicted in Figure 3, can only exhibit damped oscillations, at best, and typically solutions tend to a steady state (see Section 2). Soon after its discovery in the early ‘90s, it became evident that p53 forms a negative feedback loop with Mdm2 [109,110,111]. Systems biologists, led by Uri Alon, formulated the first mathematical model for p53 in 2000 in order to demonstrate that oscillations, in both p53 and Mdm2 concentrations, can emerge in response to a stress signal [18]. The key observation they made was that under specific assumptions p53 and Mdm2 undergo damped oscillations after a sufficiently strong damage signal; such damped oscillations were further verified by their Western blot analysis, recall Figure 1. Not only did this work spark the interest of mathematicians, who ever since have grappled with how to best model the system and derive oscillations (as we saw in Section 2), but it also engendered a truly synergistic topic within mathematical/systems biology, in which mathematical models and experiments form their own feedback loop. This Section will focus on this feedback loop; how experiments have informed mathematical models, but, equally, how those mathematical models have fed back into the design of experiments and understanding of the biology of the p53 system.

**Figure 16:**
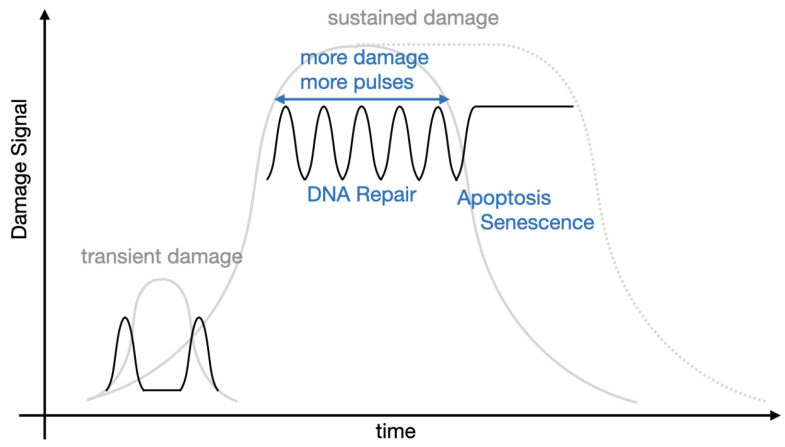
Schematic showing the role damage plays in altering p53 dynamics. Transient physiological, or low level, damage leads to pulses which are asynchronous and low frequency. Sustained damage leads to periodic pulses, the more damage being positively correlated with both the likelihood and duration of pulses. If damage persists for too long, or else cells are otherwise pushed from periodic to sustained expression, the cell enters senescence and ultimately apoptosis [16,99].

The mathematical model proposed by Alon and coauthors has only a few components but it is complicated in the sense that many different relationships are taken into consideration. Moreover, a necessary time lag between p53 activation and p53-dependent induction of Mdm2 is incorporated in the form of a hypothetical intermediary [18]. Nevertheless, the feedback from modelling flagged new experimental questions. Galit Lahav, at that time a postdoc in the group of Uri Alon and currently the chair of the Department of Systems Biology at Harvard Medical School, tried to visualise these damped oscillations of p53 and Mdm2 in individual living cells ([68], p. 100). Lahav and collaborators were the first ones to develop stable cell lines (specifically, MCF7 breast cancer cells), with the genes for p53 and Mdm2 fused respectively to cyan and yellow fluorescent proteins. The cells were exposed to γ-radiation and p53 and Mdm2 were visualised in glorious technicolor for the first time at the single cell level using time-lapse fluorescence microscopy. However, rather than damped oscillations, p53 was observed to oscillate sustainedly, although with noisy amplitude with a fixed period of about 6 h [20], see Figure 2. Lahav’s paper [20] remains an initial reference point for almost all mathematical models of p53 intracellular dynamics published ever since and Lahav’s lab is a powerhouse for p53 research. Indeed subsequent experiments from Lahav’s Lab have inspired new mathematical models and, owing to her background in systems biology, many of her subsequent papers use mathematical modelling [15,16,19,82,112,113]. These models are fully validated by building-in information from wet experiments and, importantly, have been verified against data. These studies by Lahav and coworkers have truly elucidated the dynamics of p53 in single cells in great detail, although aspects of p53 signalling still remain to be resolved due to the sheer complexity of the p53 network. In particular, it was observed that non-stressed cells show asynchronous, spontaneous p53 pulses with the same amplitude and width of pulses as for those oscillatory pulses in response to the DNA damage [99]. It is now believed that a shift from random pulses into sustained oscillations happens in individual cells under certain stress conditions (shown experimentally by [99] and mathematically by [114]), rather than a shift from a low steady state level into sustained oscillations, as per [115]. Moreover, p53 pulses are positively correlated with the amount of damage: the higher the extent of damage, the more cells exhibit p53 pulses with a detectable frequency, and the pulsatile dynamics persists for longer [99]. In Figure 16 we provide a schematic which illustrates our current understanding of how DNA damage affects the dynamics of p53 based on the experimental results of Lahav and colleagues. We note that this provides an update to the current state-of-the-art understanding with developing the reasoning from [115].

### 3.2. Mathematical Models Drive New Insights into p53 Signalling

As reviewed in the previous sections, most mathematical studies of p53 dynamics in individual cells focus on the underlying mechanisms behind the p53 oscillatory response to DNA damage. They may depend solely on mathematical modelling, or combine mathematical modelling with qualitative/quantitative experimentation. As we have seen, early mathematical models such as [19,100,102], focussed on the principles behind oscillations and laid the foundations for future models. In contrast, later mathematical models have been built on top of a core p53-Mdm2 oscillator, and include, amongst other things numerous downstream targets of p53, to achieve profiles of pro-survival and pro-apoptotic proteins in the modelling of cell fate decision.

Mathematical models may be used to further reveal, so far unknown aspects of p53 signalling, as well as crosstalk between the p53 pathway and pathways of other regulators, activated in response to genotoxic stress. Batchelor et al. investigated p53 signalling in response to UV [15]. They discovered that p53 responds with a single peak (pulse) of expression, modulated by an increase in UV dose, contrasting with the behaviour of p53 in response to DNA damage through γ-radiation. In order to understand why the same protein would behave differently under these two conditions they turned to mathematical modelling, and posited that a single missing edge in the regulatory network (specifically the absence of Wip1 ATM inhibition) could change the dynamics of p53; matching their experimental data for UV response [15] without that edge, and their experimental results following γ-radiation [82] with that edge.

Konrath and coworkers [116] combine quantitative time-lapse imaging with mathematical modelling and identify potential interactions between the p53 and NF-κB pathways. It is known that NF-κB, as a transcription factor, controls cell survival and the immune response to infection. As expected, the pulsatile behaviour of p53 is observed following DNA damage, when neither p53 nor NF-κB activity is modulated. However, when NF-κB signalling is altered, by inhibiting one particular regulator of NF-κB activity with a pharmacological inhibitor, the authors observe, experimentally, a delayed pulse peak timing and significantly longer inter-peak interval in the p53 oscillations in response to genotoxic stress. In order to reveal potential crosstalk between the pathways, the authors propose a so-called subpopulation-specific mathematical model, which is calibrated against time-resolved single cell data of the dynamic response of p53 to DNA damage, with unperturbed NF-κB signalling, and further reflects the relevant characteristics of Mdm2, Wip1 and ATM dynamics on the population level. Using this model, the authors identify several reactions in the p53 pathway (including basal and ATM-mediated degradation of Mdm2 and Mdm2-mediated degradation of active and inactive p53) which are simultaneously affected by NF-κB signalling, and they suggest that this combined action is necessary to explain altered behaviour of the p53 pathway. Thus, in order to produce meaningful hypothesis, Konrath and collaborators [116], successfully combine efforts from dry and wet experiments; in particular, they build their approach on top of three, rather general, but essential keystones: identify candidates for suitable molecular perturbations, obtain time-resolved quantitative measurements and devise an appropriate modelling strategy.

### 3.3. Application of p53 Modelling in Cancer Treatment

No paper on p53 would be complete without mentioning cancer. Certainly a frequent feature of p53 experiments and mathematical models has been to identify suitable targets for cancer therapy. Indeed, several protocols and p53-based anti-cancer therapies have been developed and already used in clinical trials. These include retrovirus- or adenovirus-mediated gene therapy to restore p53 function lost due to mutations; targeting p53-deficient cells with modified adenoviruses; pharmacological modulation of p53 protein functions; identification and design of small-molecule inhibitors of the Mdm2-p53 interactions such as nutlins or selective Mdm2 inhibitors CGM097 and HDM201 [117,118]. However, as yet there are no clear clinical studies confirming successful applications of the therapies in the treatment of cancers. In addition, the described therapeutic efforts were accompanied with unwanted side effects in normal tissues: appearance of p53-resistant tumours or premature aging, for example [119]. Thus, optimisation of doses and time of treatment, application of combined therapies, and efficacy of the treatments with small-molecule inhibitors became of great interest.

Optimisation of drug administration is perhaps one of the biggest challenges in developing anticancer therapies, for example, prolonged daily administration of some drugs over several days may result in poor tolerability [120]. To determine optimal dosing of a nutlin RG7388, a pharmacokinetics-pharmacodynamics (PK/PD) model was developed and calibrated against preclinical data obtained for selected dosing schedules in an osteosarcoma xenograft model in mice in [121]. The plasma PK part of the model is a 1-compartment ODE model, which incorporates nonlinear Hill type function for drug metabolism and first-order rate equations for drug absorption into a central compartment (tumour tissue), as well as for drug elimination from the compartment. A delay of drug effect is translated into a system of coupled differential equations, one of them modelling change of tumour volume in time. The PK/PD modelling suggested several possible intermittent clinical dosing schedules of RG7388, all confirmed as feasible options, from which weekly and daily for five days protocols have been further selected for clinical testing.

Extensive PK/PD modelling is used in [122] to improve the dosing of yet another inhibitor of p53-Mdm2 interaction, CGM097, in order to predict patients with higher risk to develop thrombocytopenia; haematologic toxicity with delayed thrombocytopenia is a well-known side effect of Mdm2 inhibitors. The study shows that the CGM097 based treatment reactivates p53, at therapeutically relevant doses of CGM097, and suggests that highly specific scheduling is required to mitigate the risk of severe thrombocytopenia. Pharmacokinetics-pharmacodynamics modelling thus takes a special place among mathematical models with practical output for clinicians and pharmacologists. At the cell population level (i.e., tissues, organs, whole human body), PK/PD modelling has been broadly used (two such examples have been given here, many others can be found in the literature) to fully describe absorption, distribution, metabolism, excretion and toxicity of anticancer drugs. Considering that individual cells are the actual targets of drug administration it may be indeed helpful to include processes that appear in individual cells after DNA damage [123], such as those processes involving p53 briefly discussed in the previous sections.

## 4. Conclusions

In almost all attempts to model p53 intracellular dynamics, many simplifications have to be made, and much biological information has to be excluded. Biomathematicians and mathematical modellers often face the question of what is the “right” information to include in a model? For example, as we have seen in Section 2.3 a couple of models such as [100,107,108,124] use the p53-PTEN-Akt-Mdm2 positive feedback loop in their oscillatory circuits. Recall, in this pathway, PTEN as a transcription target of p53 negatively regulates Akt kinase. According to some studies, Akt phosphorylates (and hence activates) Mdm2 at the serine residues 166 and 186 [125,126,127], however, phosphorylation of the latter site was not observed in [128,129]. It may be, that after Akt-dependent phosphorylation, Mdm2 shuttles from the cytoplasm to the nucleus where it downregulates p53 [125,126]. While these two reports [125,126] show a clear cytoplasmic localisation of Mdm2 when Mdm2 is not phosphorylated by Akt, other groups have shown that Mdm2 is localised in the nucleus irrespective of the Akt-dependent phosphorylation [127,128]—this is perhaps a surprising discrepancy given that similar cell lines and procedures were performed in these experiments. Moreover, post damage transcriptional activation of PTEN is not observed in [130] either. This serves to show that such mechanisms are still not readily understood by the p53 community.

Another contradiction can be found, for example, in the interpretation of ATM-dependent phosphorylation of Mdm2 at Ser395. Experimental and computational works of Batchelor et al. [15,82] and Mönke et al. [130] suggest that Mdm2 undergoes rapid degradation after being phosphorylated by ATM, and that such phosphorylation contributes to the nuclear accumulation of p53, in response to DNA damage, as well as to the mechanism for spontaneous pulses of p53. On the other hand, Gajjar and collaborators [98] show that Mdm2 changes its conformation after phosphorylation by ATM at the very same site Ser395 and, as such, Mdm2 steps out of its role as p53’s negative regulator and, instead, initiates a positive feedback arm in which p53 synthesis is stimulated, resulting in p53 molecules that are more stable in the presence of Mdm2, as already briefly discussed in Section 2.2. In both cases, works of Batchelor and coworkers, and Gajjar and coworkers, confirm accumulation of p53 after ATM-dependent phosphorylation of Mdm2; however, this accumulation appears due to the opposing fates of Mdm2. Whether it is rapid degradation of Mdm2, or a change of Mdm2’s role from negative to positive regulator of p53 (or a combination of both) has yet to be elucidated.

These two examples and apparent inconsistencies in experimental data point to the value that mathematical modelling can offer this area of research, but pose an important modelling question: simplification or clarification? Which biological processes should be included in the modelling? One can consider all the events hitherto described, even those which appear to oppose each other, and construct a large model (large in the number of parameters). Such an approach is likely to lead to “overfitting”, one can fit whatever experimental data is available with a sufficiently large parameter model, which may lead to false model interpretations and wrong conclusions. Or, one can focus on model reduction and simplification; parameter sensitivity analysis can perhaps point to obvious extraneous model terms, while common generic network motifs can be incorporated (positive/negative feedback and delays, for example). Indeed Toettcher et al. lead by example, by introducing what they call a “synthetic-natural oscillator”; a model in which core aspects of the network are modelled in full while periphery interactions are distilled into artificial terms [113]. The issue then is one of re-inventing the wheel. Do we need newer models, is there really anything they add? We are still, perhaps, in no position to answer these questions fully.

It is perhaps fair to say that the technologies to study oscillations in single cells remain quite limited and this simple fact is why we still do not have the full picture of the dynamical behaviour of p53 under normal versus stressed conditions. Since there are several ways one can view oscillations, mathematics can certainly help to fill some of the gap. It is perhaps important to identify as many oscillators as possible in a particular pathway, to try to understand, from both experimental and mathematical points of view, if and how these different factors interact with each other, what their individual functions in the oscillations are, and how they communicate. Do they have the same target, or do they act on several targets in the same pathway? It is conjectured that there must be some limitations to the cellular repertoire, in terms of which mechanisms are in place to generate an oscillating feedback system. How many of these are required as a minimum to achieve a tightly regulated cell biological response?

Over the last 20 years a wealth of study into the dynamics of p53 has permeated the literature. The purpose of this review for this special edition *“The Functional Landscape of p53”* was to offer a snapshot of the different mathematical mechanisms, which provoke oscillatory p53 dynamics, as well as commenting on the interplay between mathematical modelling and experiments in this lively field. Although every effort has been made to provide a comprehensive survey of the literature, the topic of p53 has garnered much interest from experimentalists and mathematicians alike. For additional reviews of the extensive literature please see [131,132,133].

## Appendix A. Parameters and Vector Fields for Models (1)–(4)

In the following table we provide the parameters used in the simulations of models (1)–(4). Note that the Matlab and FreeFem++ scripts to run these simulations are available as part of this publication [69].

**Model**	**Parameters**
Negative feedback model (1)	$k_{s}=2$; $k_{1}=2$; $K_{1}=0.1$; $k_{2}=2$; $K_{2}=1$; $d_{x}=d_{y}=1$; n=2
Time delay model (2)	$k_{s}=2$; $k_{1}=2$; $K_{1}=0.1$; $k_{2}=2$; $K_{2}=1$; $d_{x}=d_{y}=1$; n=2; τ=3
Spatial model (3)	$k_{s}=2$ *; $k_{1}=5$; $K_{1}=0.1$; $k_{2}=2$; $K_{2}=1$; $k_{tra}=5$; $d_{x}=d_{z}=0.1$; $d_{y}=1$; n=2; $D_{x}=D_{y}=D_{z}=0.1$; permeability for *x*, *y* and *z* is set to 10
Positive feedback model (4)	$k_{s}=2$; $k_{p}=5$; $K_{p}=1$; $k_{1}=5$; $K_{1}=0.1$; $k_{2}=2$; $K_{2}=1$; $d_{x}=d_{y}=1$; n=m=2
* The synthesis rate of p53, $k_{s}$, in the spatial model is the rate at which new p53 molecules are produced in the *whole* cell per a unit of time.	

Figure A1 contains the vector field plots for models (1), (2) and (4).

## Appendix B. A Novel Model for p53

Here we present a novel model for the regulation of p53 as per Gajjar et al. [98]. In response to damage ATM phosphorylates Mdm2 allowing it to bind with p53 mRNA; the p53 mRNA-Mdm2 interaction stimulates p53 synthesis and suppresses Mdm2-mediated p53 degradation by disrupting the p53-Mdm2 protein-protein interaction. Simply, ATM switches Mdm2 into a positive regulator of p53 by promoting p53 synthesis and inhibiting the negative regulation of p53 by Mdm2. When the damage signal is removed, ATM is no longer active and instead Wip1 reverses the phosphorylation of Mdm2 preventing the binding of Mdm2 with p53 mRNA; Mdm2 once again becomes an inhibitor of p53 by binding with its protein, promoting its polyubiquitination and degradation. These processes are distilled into the schematic given in Figure A2. We note that the induction of the p53 mRNA-Mdm2 interaction critically takes place in the nuclear compartment with ATM activity promoting Mdm2 SUMOylation bringing Mdm2 into the nucleus, as such spatial aspects are a key component of this model although not explicitly depicted in the schematic given below. By the same token there are other interactions such as the downstream upregulation of Wip1 by p53 that are not present in the schematic but may be present in the model.
